# Louis Pasteur: A Legacy Unmasked

**DOI:** 10.7759/cureus.68080

**Published:** 2024-08-29

**Authors:** Pankaj C Jambholkar, Sonali G Choudhari, Mayank Sharma

**Affiliations:** 1 Community Medicine, Jawaharlal Nehru Medical College, Datta Meghe Institute of Higher Education & Research, Wardha, IND

**Keywords:** public health, pasteurization, fermentation, sterilization, bacteriology, historical vignette

## Abstract

Louis Pasteur is perhaps the most globally recognized French scientist. His groundbreaking discoveries in molecular chirality and advancements in fermentation greatly benefited brewers and winemakers. Pasteur introduced the process of pasteurization to sterilize wines and significantly contributed to the development of germ theory, which made Joseph Lister’s antiseptic surgical techniques possible. Despite initially disproving Antoine Béchamp’s theory that silkworm disease was caused by a microbial infection, Pasteur tackled this issue effectively. Building on the work of Henri Toussaint and Pierre Victor Galtier, he developed vaccines for pig erysipelas, chicken cholera, anthrax, and rabies. Pasteur also coined the term “vaccination,” which Richard Dunning had used before Edward Jenner expanded upon it. Although Robert Koch criticized Pasteur’s vaccination methods as ambiguous, historians have clarified many of the myths surrounding Pasteur. This review explores Pasteur’s career, his undeniable achievements, and the realities behind the legendary figure who strove to make a significant impact on science and medicine.

## Introduction and background

Louis Pasteur (1822-1895) is recognized as one of the greatest scientists in history, having laid the foundation for modern medical practices through his pioneering work in microbiology, chemistry, and public health [1]. Despite his significant contributions, myths and exaggerations often cloud the true scope of his achievements. This biography aims to clarify these myths and provide an accurate portrayal of Pasteur’s impact. By applying scientific principles to practical challenges, Pasteur revolutionized public health and medicine, drastically reducing the mortality rate from infectious diseases. His enduring legacy is reflected in the continued influence of his methods and discoveries on medical science [1].

Pasteur is renowned for his groundbreaking work in chemistry and microbiology. His name is closely linked with the development of vaccines, the pasteurization process, and the germ theory of disease - achievements that have profoundly shaped the scientific community and public health. Pasteur’s contributions have elevated him to near-mythical status, often depicted as a singular hero who revolutionized medicine and microbiology [2].

## Review

Early life and education

**Figure 1:**
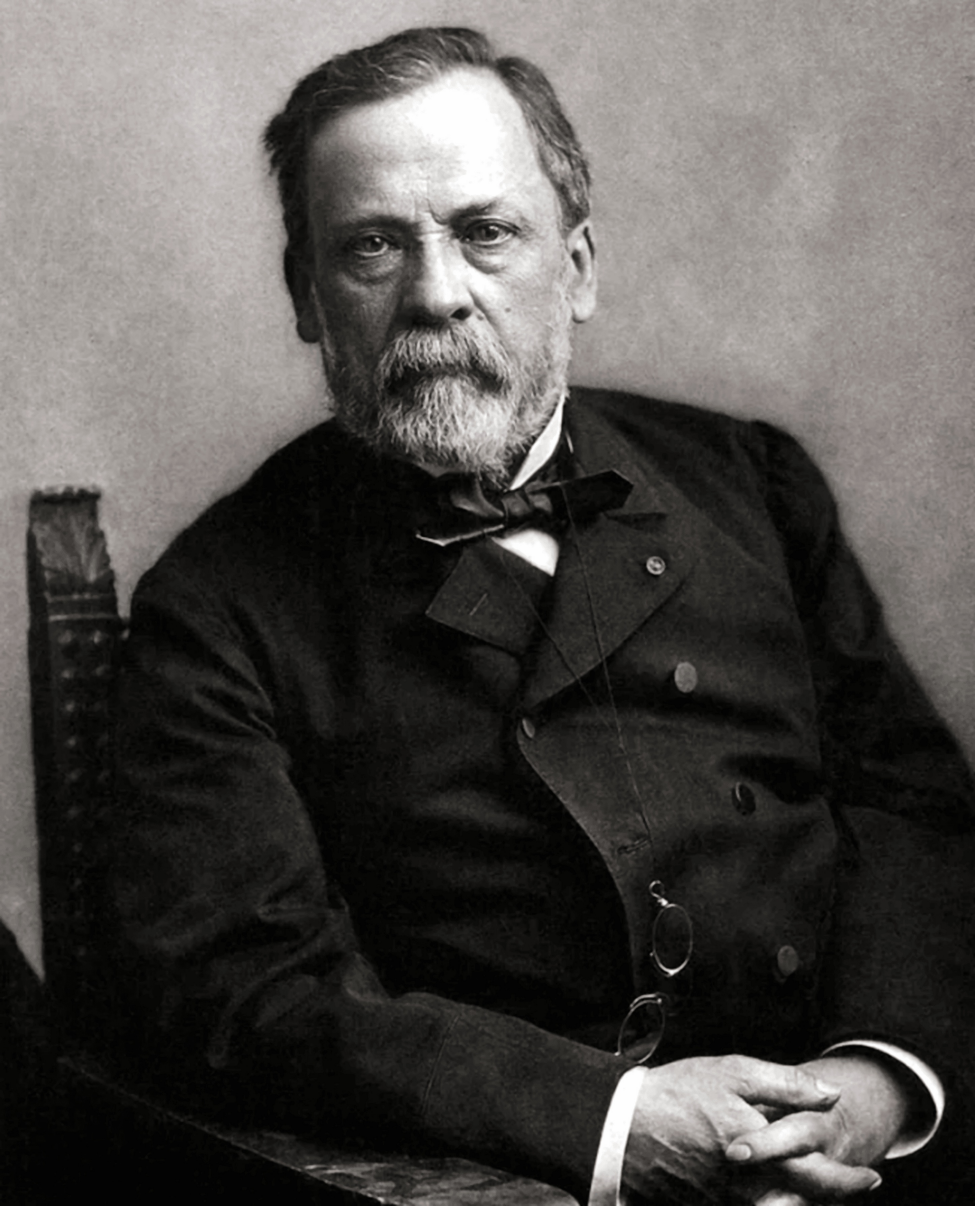
Studio portrait of Louis Pasteur, restored Source: File: Louis Pasteur, photograph by Paul Nadar (1856-1939)

Louis Pasteur was born in Dole, France, and was raised in a humble family (Figure 1). His early interest in art and science led him to pursue education at the École Normale Supérieure in Paris, focusing on chemistry [3]. This background likely instilled in the younger Pasteur the strong patriotism that later became a defining element of his character. Although often seen as a child prodigy, Pasteur was an average student whose scientific skills developed through persistent effort. He showed early interest in science, attending primary school in Arbois and earning a Bachelor of Arts at the Collège Royal in Besançon [4].

In 1842, Pasteur enrolled at the École Normale Supérieure in Paris, earning a doctorate in chemistry in 1847. Contrary to Aristotle’s long-held belief that life arose from non-living matter through a “vital force,” Pasteur’s work disproved this idea. Spallanzani and Pasteur together challenged the spontaneous generation theory [5].

Pasteur’s research

Louis Pasteur’s research revolutionized microbiology by demonstrating that microorganisms are responsible for fermentation and spoilage, leading to the development of the pasteurization process, which kills harmful bacteria in food and drinks. His work on germ theory became foundational to modern medicine. While investigating a beer fermentation issue in Lille, Pasteur discovered bacteria and an optically active component, amyl alcohol, confirming that fermentation was driven by living organisms. In 1857, he published a seminal work on lactic fermentation, marking a pivotal moment for germ theory [6]. He also explored butyric acid fermentation, further advancing the field.

A common myth is that Pasteur independently conceived and developed all his scientific ideas and implementations. In reality, he built upon the contributions of earlier scientists, such as Lazzaro Spallanzani and Antoine Béchamp, as well as his contemporaries. Pasteur’s brilliance lay in his ability to combine existing knowledge with meticulous experimentation to validate his theories. He notably disproved Aristotle’s ancient belief that life could spontaneously arise from non-living matter due to a “vital force” or “vital heat.” Alongside Spallanzani, Pasteur effectively challenged this long-held theory of spontaneous generation [7,8].

Pasteur’s life and career

Pasteur is one of the most renowned scientists globally, celebrated for his substantial contributions to humanity. His name is synonymous with groundbreaking concepts such as germ theory, vaccination, and the discovery of “life crystals.” Despite his humble beginnings, there was little indication that Pasteur would emerge as a pioneer in biology and medicine. His detailed notes on silkworm epidemics enabled him to demonstrate the critical role of specific germs in contagious diseases [9].

Pasteur’s development of the fowl cholera vaccine is often regarded as the foundation of immunology. He eventually gained worldwide recognition for his pioneering work on the anthrax and rabies vaccines, which solidified his legacy as a key figure in the advancement of medical science [10].

Vaccination and public health

The rabies and anthrax vaccines developed by Pasteur were significant in illustrating the fundamental concepts of immunization. In particular, his rabies vaccine saved many lives and solidified immunization as a cornerstone of disease prevention [11]. Pasteur’s representation of his discoveries and accomplishments has additionally caused historical controversy. Even with his revolutionary findings, some of his research, particularly the ones involving animals, would fail ethical scrutiny. Moreover, Pasteur sometimes overstated the significance of his findings and underestimated the contributions of his collaborators and colleagues. Louis Pasteur’s creation of vaccines for anthrax and rabies was instrumental in showcasing the power of immunization and its role in disease prevention. His rabies vaccine, in particular, was a landmark achievement that saved countless lives [4,12].

Personal life and legacy

Pasteur was deeply committed to using his scientific knowledge for the betterment of society. He founded the Pasteur Institute in Paris, which became a world-renowned research center specializing in microbiology and infectious diseases. Contrary to the myth of Pasteur as a solitary genius working in isolation, his achievements were supported by a dedicated team of students, collaborators, and family members. Among them, his wife, Marie Pasteur, played a crucial role not only in managing the laboratory but also in supporting his work in more substantive ways. Her contributions were instrumental to Pasteur’s success and extended far beyond mere administrative duties [13].

Pasteur’s techniques were immediately adopted

Pasteur’s techniques, like pasteurization and sterilization, took time to be adopted in surgical practices due to resistance and slow knowledge spread. Opposition from traditionalists and the sluggish spread of scientific information resulted in his techniques taking years, even decades, to be widely adopted. Lister’s use of antiseptic methods from Pasteur’s work also faced initial opposition before gaining acceptance [14].

Pasteur was universally celebrated during his lifetime

Pasteur encountered significant resistance and controversy throughout his professional life. His questioning of accepted medical and scientific ideas, like spontaneous generation, sparked notable opposition from certain peers. His competitive spirit and public disagreements with fellow scientists, including Antoine Béchamp, also resulted in professional rivalries and disputes [5]. Nevertheless, his determination and the eventual triumph of his theories garnered him broad acknowledgment and admiration.

Pasteur’s influence on Lister

Lister sought to lower the rate of wound sepsis that usually occurs in hospital settings. He became fascinated by Louis Pasteur’s work after discovering it via Glasgow chemistry professor Thomas Anderson (1819-1874) [13]. Pasteur deduced that airborne germs produced fermentation and putrefaction in liquids such as milk and juice. His studies refuted the widely held belief that dangerous bacteria developed independently. Pasteur had a big impact on Lister [14].

In a packed amphitheater at the Sorbonne in Paris on December 27, 1892, the scientific community, including Lister, celebrated Pasteur’s 70th birthday. Attendees included scientists, Institute de France representatives, ambassadors, university employees, and state ministers. Ten years later, Lister is seen climbing the steps to congratulate Pasteur in a painting by Jean-André Rixens [15]. Encouraged by Pasteur’s findings, Lister hypothesized that airborne pathogens were the cause of wound infection and suppuration. Lister believed at the time that air in medical environments needed to be cleaned since it was difficult to discern between benign and hazardous microorganisms [15]. Lister proposed a method to prevent infections in the postoperative setting: establishing an antiseptic barrier between the air surrounding the incision and the wound. It was even proposed that eliminating or destroying airborne bacteria with heat, chemicals, or filtration may prevent all diseases [14].

## Conclusions

Louis Pasteur significantly influenced microbiology, chemistry, and public health with his pasteurization, germ theory, and vaccine discoveries. However, his achievements were not solely his own; they resulted from careful experimentation, collaboration with peers, and the scientific milieu of his time. The heroic narrative often obscures the communal nature of scientific progress and the ethical complexities Pasteur faced. A balanced view of his legacy acknowledges his brilliance and his work’s collaborative, ethical, and cultural context. Celebrating Pasteur in this way honors his contributions and the broader scientific community’s impact on modern science.
